# One Health genomic surveillance reveals structured urban rabies transmission and major surveillance gaps

**DOI:** 10.64898/2026.07.07.736726

**Published:** 2026-07-10

**Authors:** Kirstyn Brunker, Alejandra Dávila-Barclay, Elvis W. Díaz, Sandeep Kasaragod, Rowan Durrant, Katie Hampson, Edith Zegarra, Ynés Monroy, Ricardo Castillo-Neyra

**Affiliations:** 1 School of Biodiversity, One Health & Veterinary Medicine, University of Glasgow, Glasgow, United Kingdom; 2 MRC-University of Glasgow Centre for Virus Research, University of Glasgow, Glasgow, United Kingdom; 3 One Health Unit, School of Public Health and Administration, Universidad Peruana Cayetano Heredia, Lima, Peru; 4 Department of Biology, McMaster University, Hamilton, Ontario, Canada; 5 Laboratorio de Referencia Regional de la Gerencia Regional de Salud de Arequipa, Arequipa, Peru; 6 Department of Biostatistics, Epidemiology & Informatics, Perelman School of Medicine, University of Pennsylvania, Philadelphia, Pennsylvania, USA; 7 Department of Pathobiology, School of Veterinary Medicine, University of Pennsylvania, Philadelphia, Pennsylvania, USA

**Keywords:** Genomics, Nanopore, One Health, Outbreak, Rabies, RABV, WGS, Zoonosis

## Abstract

Persistent local foci remain a barrier to eliminating dog-mediated rabies. The processes sustaining micro-scale transmission, particularly in complex urban systems, remain poorly understood. We apply an integrated One Health genomic epidemiology framework to reconstruct a decade-long rabies virus (RABV) epidemic in Arequipa, Peru. Combining 133 new whole genomes with existing data, we produce the most comprehensive canine RABV dataset in Latin America and use whole-genome-informed phylogenetic, phylodynamic, and landscape analyses, to trace the epidemic from its first detection in 2015. Transmission was dominated by a single lineage estimated to be introduced around 2012, which spread for approximately 3 years before detection. We find that only 1–2% of infections are routinely detected, revealing extensive cryptic transmission and undermining case-based metrics for verifying disease freedom. Additional regional and transboundary introductions were detected, but only one resulted in sustained transmission. Within Arequipa city, transmission is highly structured, concentrated in densely populated and socioeconomically deprived areas, and shaped by urban connectivity, with roads and dry water channels facilitating spread and rivers acting as partial barriers. Together, our findings demonstrate that rabies persistence reflects interacting processes across spatial scales and support genomic-informed, spatially targeted surveillance and control strategies.

## Introduction

Rabies is a fatal zoonosis responsible for tens of thousands of human deaths annually, despite the availability of effective vaccines for both humans and animals^1,2^. Most human cases result from dog-mediated transmission, and global efforts are underway to end human deaths from canine rabies by 2030^3^. Substantial progress has been made across Latin America through coordinated mass dog vaccination campaigns that have drastically reduced disease burden^4^. However, elimination remains incomplete, with persistent transmission in several regions and continued risk of re-emergence in areas previously declared rabies-free^5–8^.

Urban and peri-urban rabies virus (RABV) persistence presents a particular challenge to elimination. In contrast to broader spatial scales, where viral populations are strongly structured by geographic barriers and host associations, urban environments exhibit high host density, heterogeneous infrastructure, and frequent human-mediated movement, facilitating viral admixture through co-circulating lineages and overlapping transmission chains^9–14^. Together, these features generate spatially heterogeneous dynamics that sustain cryptic endemic circulation and obscure viral transmission pathways and evolutionary processes. Consequently, conventional surveillance approaches, which rely largely on the timing and location of detected cases, provide only a partial view of transmission dynamics, making it difficult to identify the mechanisms sustaining persistence.

Advances in whole-genome sequencing (WGS) and genomic epidemiology have transformed the study of RABV, providing insight into the processes that shape viral spread and persistence^13,15–18^. By integrating genomic and epidemiological data, these approaches have identified key mechanisms underlying transmission across spatial scales, including how geographic distance and landscape barriers structure viral diffusion at regional and continental scales^17,19–21^ and the influence of host contact networks, population density, and reintroduction events at finer scales^12–14^. Despite these advances, few studies have resolved transmission at the spatio-temporal scales required to inform urban rabies control, and the translation of genomic insights into actionable surveillance and intervention strategies remains limited^22^.

In regions approaching elimination, such as Latin America, this gap is especially important. As incidence declines, surveillance systems face new challenges^23^, including detecting low-level persistence, identifying sources of re-emergence and distinguishing between local transmission and repeated introductions. These questions are difficult to resolve using conventional epidemiological data alone but are central to achieving and sustaining elimination. In this context, sequence data is especially powerful when integrated with spatial, environmental, and intervention data within a One Health framework^16,24,25^, as well as providing a powerful complementary and independent line of evidence^26^, to reconstruct transmission histories^19,21,27–29^, identify introduction events^16,24,30^, and estimate epidemic size^31^. Despite this potential, genomic surveillance remains largely absent from routine rabies control and surveillance across much of Latin America, with only one recent study (our previous work)^30^ publishing contemporary WGS data. A scarcity of data limits the resolution of genomic inference needed to inform interventions.

**Figure 1.**
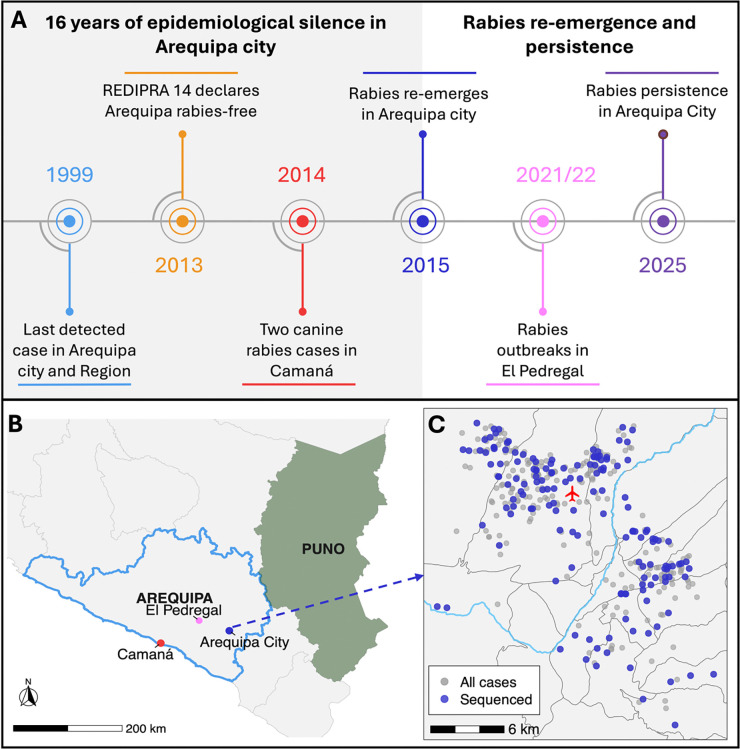
Epidemiological timeline and spatial context of rabies elimination, reintroduction, and persistence in Arequipa, Peru. (A) Timeline of canine rabies in Arequipa showing 16 years of epidemiological silence following the last detected case in 1999 and declaration of rabies-free status in 2013, followed by re-emergence in 2014–2015 and persistence through to 2025. (B) Map of the study area showing Arequipa region (blue outline) and key locations implicated in rabies events. (C) Spatial distribution of reported rabies cases in Arequipa city, with the Chili River and Rodríguez Ballón International Airport indicated for spatial context. Grey points show all reported cases; blue points indicate sequenced samples, with grey polygons indicating administrative districts.

The re-emergence and endemicity observed in Arequipa, Peru, over the past decade exemplify this gap, highlighting how poorly resolved the drivers of persistence remain. The city and region of Arequipa were included in the 14th Meeting of Rabies Programs Directors of the Americas (REDIPRA) statement declaring 88% of Peruvian territory free of dog-mediated rabies by 2013^7,32^, following 14 years of epidemiological silence at the time of the declaration^33,34^; however, transmission was detected again in the Arequipa Region in 2014 and in the city of Arequipa in early 2015^35^ (Figure 1). Over the subsequent decade, transmission persisted, with approximately 400 laboratory-confirmed canine rabies cases detected^6^. This persistence occurred despite intensified control efforts including mass dog vaccination and reactive outbreak response, albeit at varying frequency^5,36–38^. Detailed epidemiological and One Health investigations identified important mechanisms of spread, including movement along urban water channels that are dry for most of the year^10^, expansion into peri-urban areas, and associations with socio-economic status^39^. However, these inferences are largely derived from surveillance and modelling-based analyses. While informative, these approaches alone cannot disentangle the multiple processes that generate observed epidemic patterns^27,40^. As a result, key uncertainties remain, including the timing and source of introduction(s), the extent of undetected transmission, the relative importance of local versus long-distance spread, and how control strategies can be optimised to interrupt transmission.

In this study, we applied an integrated genomic epidemiology framework to reconstruct a decade-long canine rabies epidemic in Arequipa, Peru, providing insights into RABV origins, transmission dynamics, and the mechanisms underlying viral persistence.

## Methods

The study protocol (No. 216867) was approved by the Institutional Ethics Committee for the Use of Animals (IECA) of the Universidad Peruana Cayetano Heredia (CERTIFICATE-IECA-021-04-25).

### 1. Study area and epidemiological context

The city of Arequipa is located in the Southern Peruvian Andes within the Arequipa Region, bordering the Puno Region, which shares a boundary with Bolivia. Arequipa city comprises a central urban core surrounded by expanding peri-urban settlements arranged in a ring of younger neighbourhoods. The Chili River bisects the city into north-west and south-east sectors, linked by multiple pedestrian and vehicle bridges, with an associated water channel system extending throughout the urban area. The last reported rabies case in the city occurred in 1999^33,34^. Following a prolonged period of epidemiological silence, the region was included among areas considered rabies-free during a 2013 evaluation under REDIPRA, a Pan American Health Organisation-led initiative^41^, at which time approximately 88% of Peruvian territory was classified as rabies-free^32^ (Figure 1). However, in 2014, two canine cases were reported in Camaná^42^, a coastal city in the Arequipa Region, and subsequently, in March 2015, a dog rabies case was confirmed in the city of Arequipa, marking the first documented reintroduction in urban Latin America. Between 2015 and March 2025^35^, canine rabies spread across the city, with cases detected in nearly all city districts and occasional transmission events in neighbouring city, El Pedregal^30^. Early transmission was associated with movement along the city’s water channels^9,10^ while later spread reflected sustained urban circulation across interconnected neighbourhoods. Control efforts primarily relied on mass dog vaccination campaigns with variable spatial and temporal coverage^36,43^, alongside outbreak response activities including ring vaccination, epidemiological investigation, and contact tracing following case detection.

### 2. Surveillance system and case data

Since the reintroduction in 2015, a total of 398 animal cases have been reported in Arequipa between 2015 and 2025, including 12 dog cases in El Pedregal and 386 cases in Arequipa city (383 dogs, 3 cats). Only one human death from rabies in 2023 has been recorded. The Arequipa-Caylloma Health Network oversees rabies surveillance within the city, following established reporting and response protocols^38,44^ and submitting brain samples to the Arequipa Regional Reference Laboratory (LRR). Throughout the epidemic, the laboratory has also received samples from private clinics and veterinary professionals, as well as from the research team during support of outbreak control activities^45^. Surveillance data were obtained from LRR including weekly submissions with corresponding diagnostic results. Diagnostic testing was performed using Direct fluorescent antibody (DFA) testing according to national guidelines^46^. Sample duplicates were confirmed at the Peruvian National Institute of Health (INS), with discrepancies resolved using LN34 RT-PCR^47^ or mouse intracerebral inoculation^44^ when available.

### 3. Sample processing and sequencing

Laboratory-confirmed rabies-positive brain samples in DNA/RNA Shield were provided by the LRR for downstream processing, along with associated epidemiological metadata. Samples were complemented with detailed case investigation data collected during outbreak response activities, including dates of symptom onset, death, and reporting, as well as location (district and geographic coordinates), travel history, and descriptive case narratives.

Total viral RNA was extracted from DFA-positive brain samples using the Quick-RNA Viral kit (Zymo Research, Irvine, CA, USA) following manufacturer instructions and stored at −80°C before downstream processing. RNA was reverse-transcribed and amplified using RT-PCR with a Peru-specific RABV primer scheme (Pedregal paper), following established rabies sample-to-sequence protocols^48,49^. Libraries were prepared using a native barcoding kit (SQK-NBD114.24), with negative controls included in each sequencing run. Libraries were sequenced on R10 flow cells using a MinION platform. Consensus sequences were generated using an established RABV bioinformatics pipeline^48^. A total of 133 high-quality genomes were generated and combined with previously published sequences from Arequipa, El Pedregal and Puno^30^, resulting in a dataset of 167 Peruvian WGS.

### 4. Phylogenetic and phylodynamic analyses

Phylogenetic and phylodynamic analyses were conducted across three nested spatial scales to (i) place Arequipa viruses within regional diversity, (ii) reconstruct evolutionary relationships among Peruvian lineages, and (iii) resolve fine-scale transmission dynamics within Arequipa.

#### 4.1 Regional-scale context

To contextualise Peruvian RABV sequences within broader regional diversity across Latin America and the Caribbean (LAC) and to identify the dominant lineage circulating in Arequipa, a comprehensive alignment of LAC canine RABV sequences was assembled. Publicly available sequences (>200 bp), together with an outgroup representing the Cosmopolitan Africa 4 clade (GenBank: KF154998), were retrieved from the RABV-gTK database (https://rabv-gdb.cvr.gla.ac.uk/) using the V-gTK resource (https://github.com/centre-for-virus-research/V-gTK). Newly sequenced genomes from this study (n = 133) were incorporated into the alignment using MAFFT v7.520, preserving alignment length. A maximum-likelihood phylogeny was inferred using IQ-TREE under a GTR+Γ model with 1000 ultrafast bootstrap replicates. Outliers (top 1% longest terminal branches) were identified and removed using the ape package. The tree was rooted using the outgroup and visualised in R using ggtree, with geographic mapping performed using sf and rnaturalearth.

#### 4.2 Peru-scale phylogenetic inference

To determine the evolutionary relationships among sequences from Arequipa city (n = 152), El Pedregal (n = 12), and available historical WGS from Puno and Madre de Dios (n = 4), a Bayesian time-scaled phylogeny was inferred using BEAST v10.5.0^50^. Temporal signal was assessed using root-to-tip regression in Clockor2^51^, confirming suitability for this analysis (Supplementary Fig. S1). Initial exploratory runs identified a divergent sequence associated with an external introduction (ID: 219_2024) that was excluded in the final analysis due to its disproportionate effect on temporal structure, resulting in a final dataset of n=166 WGS. A GTR+Γ substitution model partitioned into coding and non-coding regions was used with an uncorrelated lognormal relaxed molecular clock and constant population size prior. MCMC chains were run for 30 million iterations, sampling every 3,000 steps. Convergence was assessed in Tracer (ESS > 200). Posterior trees were summarised using TreeAnnotator with HIPSTR and 10% burn-in removal.

#### 4.3 City-scale phylogeography

To reconstruct city-scale viral diffusion and identify environmental factors associated with transmission, all subsequent analyses focused on the dominant local lineage (n = 148).

##### i) Discrete trait analysis

Discrete trait phylogeography was conducted under an uncorrelated lognormal relaxed clock with an asymmetric discrete trait model incorporating Bayesian stochastic search variable selection (BSSVS). Markov jumps and Markov rewards were used to quantify transition rates and lineage persistence within states. Discrete traits represented epidemiological and administrative factors hypothesised to structure transmission, including position relative to the Chili River, urbanicity, and administrative districts. Administrative boundary shapefiles were obtained from the Peruvian National Institute of Statistics and Informatics (INEI; https://ide.inei.gob.pe/#capas). Urbanicity was defined using locality characteristics and local expert knowledge. Data sources for all other landscape variables are provided in Table S1. Some discrepancies between district labels and case coordinates reflect the administrative assignment of microredes (health networks), which may not always align with their precise geographic location. For the discrete analysis, we therefore retained the recorded district labels. To assess robustness to sampling bias, we downsampled the dataset (n = 148) to a more balanced subset (n = 100) using Treemmer^52^. Districts with >10 sequences (Cerro Colorado, Mariano Melgar, and Yura) were selectively reduced to limit over-representation, with sequences randomly subsampled within each district while retaining geographic coverage.

##### ii) Continuous trait analysis

Continuous phylogeographic reconstruction, using case coordinates (latitude/longitude), was performed in BEAST using a GTR+Γ model, an uncorrelated lognormal relaxed molecular clock, and a Skygrid coalescent prior. MCMC chains were run for 200 million iterations, sampling every 20,000 steps. Convergence and ESS (>200) were verified in Tracer. Spatio-temporal diffusion histories were reconstructed from posterior trees, and 100 post-burn-in trees were extracted using the R package SERAPHIM 2.0^53,54^. These were converted into phylogenetic branch-specific dispersal events across the city landscape. From these, dispersal statistics were estimated, including mean and weighted branch dispersal velocity and diffusion coefficients^55,56^.

**Figure 2.**
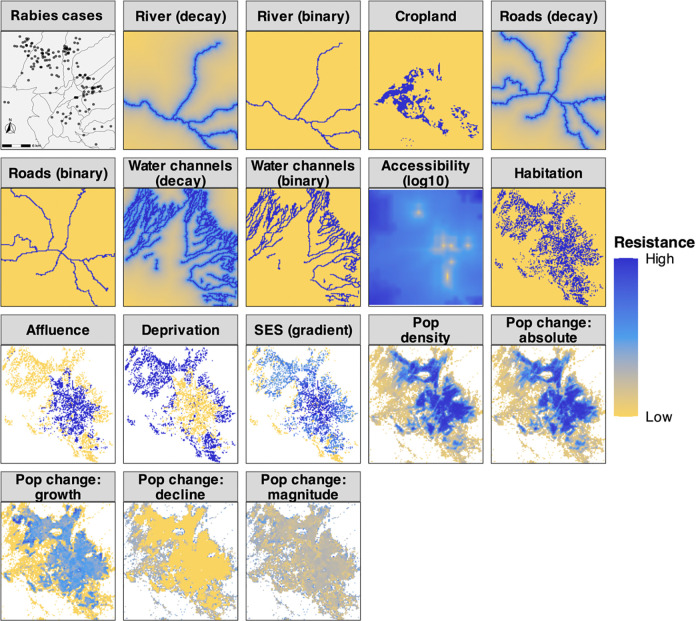
Landscape variables used to model RABV spread in Arequipa, represented as resistance surfaces. Variables include features representing movement barriers, land cover, accessibility, socio-economic context and population dynamics. The first panel shows the study area and distribution of sequenced rabies cases used in the analysis. Linear features (rivers, roads, and water channels) were represented as binary surfaces or distance-decay surfaces, where influence decreases with distance.

The same extracted branch-specific dispersal vectors were also used within the SERAPHIM framework to determine the influence of landscape variables on diffusion. The study landscape was defined as a 200m resolution grid encompassing the geographic extent of the sequenced cases with a 1 km buffer. Environmental layers included rivers, roads, water channels, built-up areas, and socioeconomic status (SES), which were all harmonised to this base raster grid (Figure 2). Each environmental variable was represented as a resistance surface, with values normalised (0–1) and scaled (k = 10, 100, or 1000), alongside a neutral (null) surface where all cells were set to 1 to represent homogeneous space. SES was represented in two ways: a continuous gradient derived from categorical classes (A–F) and rescaled (6–1) to reflect decreasing socioeconomic status, and a binary classification distinguishing affluent versus deprived areas. Linear features (rivers, roads, and water channels) were represented as binary surfaces, where influence is restricted to the mapped feature, and as distance-decay surfaces, where influence decreases with distance in 100 m intervals, capturing spatially graded infrastructure effects. More detail on the construction of landscape resistance surfaces, including data sources (Table S1), can be found in the Supplementary text.

These surfaces were evaluated under alternative model formulations, where environmental features were treated as resistances (impeding movement) or as conductances (facilitating movement) within the SERAPHIM framework. To assess the influence of environmental structure on viral diffusion, we tested whether environmental variables improved the explanation of phylogenetic movement patterns compared to a null model based on geographic distance alone. This was evaluated using SERAPHIM’s correlation-based statistic (*Q*) that quantifies the added explanatory power of environmental heterogeneity, with significance assessed against null models generated by randomisation of lineage locations across phylogenetic trees. We further evaluated environmental associations using two complementary statistics. The first (*E*) measures whether lineages are preferentially associated with particular environmental conditions at phylogenetic node locations. The second (*D*) captures directional tendencies along branches, indicating whether viral lineages move towards or away from environmental gradients. For all statistics, observed values were compared to null expectations derived from randomised trees, with support summarised using approximated Bayes factors.

### 5. Outbreak size estimation

Outbreak size and annual case numbers for Arequipa city were estimated following Durrant et al. (2026)^31^. The total tree length of the city-scale phylogenetic tree (n = 148) was combined with per-generation substitution rates derived from BEAST posterior estimates and a mean generation interval of 32 days. A nonlinear equation was solved for sampling proportion using the quadratic formula:

$$T\sqrt{p}-\mu (S-p)=0$$

where T is the tree length, μ is the per-generation substitution rate, S is the number of tips, and p is the proportion of cases sequenced. Posterior estimates of outbreak size were obtained as *S/p*, with uncertainty propagated across 9,002 posterior rate samples. Annual incidence was reconstructed by sequential pruning of the phylogeny by year, estimating cumulative outbreak size per time slice; differences between years were used to derive annual case counts.

## Results

### Genomic context and regional connectivity

**Figure 3.**
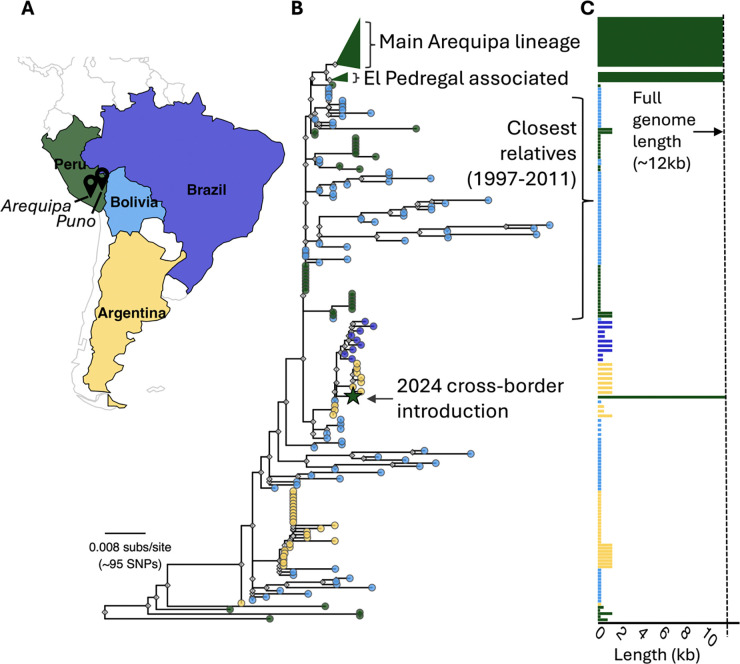
Phylogenetic context of canine RABV in Arequipa, Peru (2015–2025). (A) Geographic context of the Cosmopolitan AM5 lineage across Latin America, with countries coloured by origin and key locations (Arequipa and Puno) indicated. Only countries with available sequence data for the Cosmopolitan AM5 lineage are shown; RABV is present in additional countries across the region not represented here. (B) Maximum-likelihood phylogeny of 296 AM5 sequences, including isolates from this study, showing the placement of Arequipa RABV within a broader regional context. The dominant Arequipa and El Pedregal-associated lineages are collapsed for clarity (triangles). The closest historical relatives (1997–2011) of the Arequipa-associated RABV are indicated; Peruvian sequences within this cluster are predominantly from the Puno Region. Tip colours denote country of origin (as in A), and grey diamonds indicate nodes with ≥90% bootstrap support. A 2024 Arequipa case from a separate introduction is annotated. (C) Sequence length distribution for all taxa in the phylogeny. The predominance of shorter sequences highlights the limited availability of whole-genome data in Latin America outside this study.

**Figure 4.**
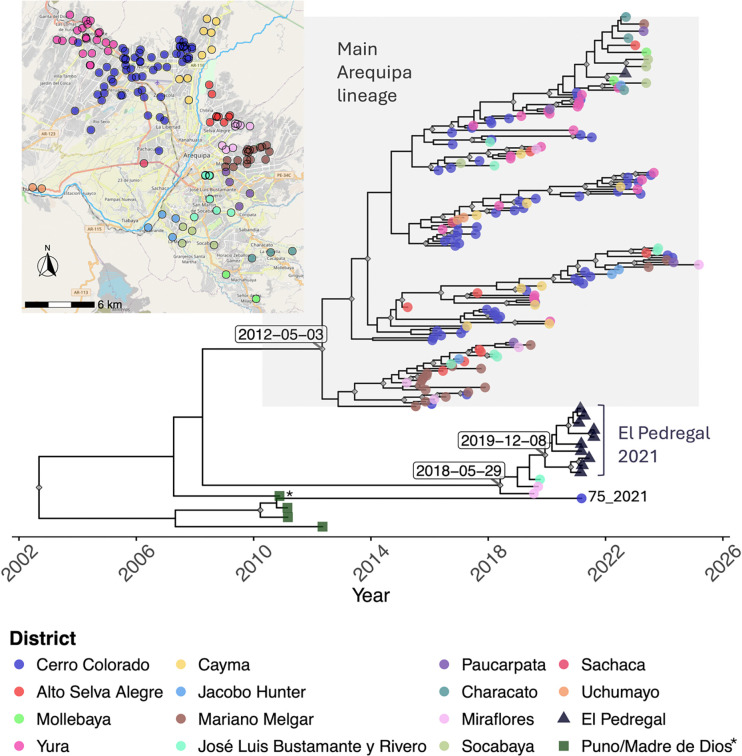
Time-scaled phylogeny of RABV genomes from southern Peru. Maximum clade credibility tree inferred from whole-genome RABV sequences collected in Arequipa city (n = 152), El Pedregal (n = 12), and historical isolates from the Puno (n = 3) and Madre de Dios* (n = 1) regions. Branch lengths are scaled in years, and internal node symbols indicate posterior support > 0.9. Tip colours denote sampling location. The contemporary epidemic in Arequipa is derived from a single dominant lineage (highlighted), with annotated ancestral nodes indicating the estimated timing of key introduction events. The inset map shows sample locations within Arequipa, coloured by district, overlaid on an OpenStreetMap basemap illustrating the heterogeneous urban landscape. Sample 75_2021, representing a putative introduction linked to the Puno Region, is labelled. Apparent mismatches between case location and districts on the map reflect the administrative assignment of microredes (health networks); recorded district labels were retained for discrete phylogeographic analysis.

A total of 133 new whole-genome RABV sequences from the city of Arequipa were generated and combined with 34 previously published genomes from Peru, yielding a dataset of 167 whole genomes. The dataset comprised 152 genomes from Arequipa city, 12 from El Pedregal, 3 from the Puno Region, and 1 from the Madre de Dios Region. This represents approximately 83% of all publicly available RABV whole genomes from LAC, highlighting the limited genomic surveillance across the region. To place these data in context, we analysed 1,816 publicly available RABV sequences (>200 bp) from LAC (Supplementary Fig. S2). Only 11% were whole genomes (n = 199, with 152 from Arequipa region), with the remainder comprising partial gene fragments or individual genes. All sequences from this study clustered within the Cosmopolitan AM5 lineage. Within the AM5 lineage, comprising 296 of the original 1,816 sequences from LAC, Arequipa sequences were most closely related to viruses from Bolivia and the Puno Region of Peru, indicating regional connectivity across the Andean corridor (Figure 2). Bolivia-derived sequences frequently occupied basal positions in the AM5 phylogeny, consistent with unsampled diversity and a potential regional source population. In addition to the dominant Arequipa-associated cluster, the tree identified multiple independent introductions into the city, including a recent case clustering with sequences from Argentina, Bolivia, and Brazil (highlighted in Figure 3), and another associated with Puno, which is more clearly resolved in Figure 4 (collapsed with the Arequipa triangle in Figure 3). However, limited genomic resolution across the region constrains precise inference of source populations.

### A single dominant lineage drives sustained transmission amid repeated introductions

Bayesian time-scaled phylogenetic reconstruction of the Peruvian dataset resolved multiple well-supported lineages but revealed that transmission in Arequipa is overwhelmingly dominated by a single lineage comprising 148 of 166 genomes (89%) (Figure 4), of which one case is from El Pedregal in 2022. This lineage was inferred to have been introduced around April 2012 (95% HPD: 2011.39–2013.85), approximately three years prior to the first detected case in 2015, and exhibited limited within-cluster diversity (mean patristic distance: 13 SNPs), consistent with sustained local transmission following a single introduction. In contrast, the four other Arequipa-associated sequences constituted two independent introductions that did not result in long-term persistence and three sequences associated with a contained El Pedregal 2021 outbreak (Figure 4).

A distinct cluster associated with the 2021 El Pedregal outbreak (n = 10) formed a well-supported clade with an MRCA dated to August 2019 (95% HPD: 2019.74–2020.71), consistent with a relatively recent local transmission event. Three genetically related sequences sampled in the city of Arequipa in 2019 were phylogenetically basal to this cluster and, when included, placed the broader clade MRCA at May 2018. This structure is consistent with introduction from broader, unsampled regional viral diversity, with potential transient circulation in the city of Arequipa prior to spatial expansion into El Pedregal, where it resulted in a contained outbreak in 2021^30^. Notably, a single El Pedregal case sampled in 2022 clustered within the dominant Arequipa lineage, consistent with a rare spillover event linking the otherwise sustained Arequipa transmission chain to El Pedregal. Although only one genome was obtained from El Pedregal in 2022, previous analyses concluded that this represented an independent introduction into the area rather than continuation of the 2021 outbreak^30^.

Two sequences (75_2021 and 219_2024) were highly divergent from both the dominant lineage and the El Pedregal cluster, supporting independent introductions. These were clearly resolved in both the whole-genome dataset and the broader contextual phylogeny, which included a high proportion of partial sequences. Sequence 219_2024 differed by 161–182 SNPs from the dominant and El Pedregal lineages, while sequence 75_2021 differed by 18–39 SNPs. These distances substantially exceeded within-lineage diversity elsewhere (Arequipa: 13 SNPs; El Pedregal: 8 SNPs), supporting their placement outside established transmission chains.

### The epidemic is substantially under-detected

**Figure 5.**
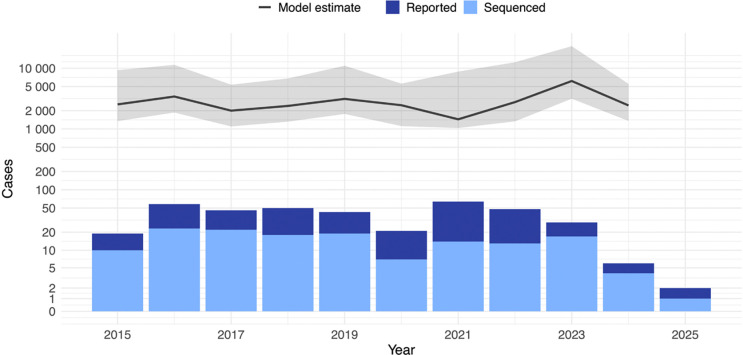
Outbreak size estimation for endemic rabies virus in Arequipa (2015–2025). Reported (dark blue) and sequenced (light blue) cases are shown alongside phylogenetically informed model estimates of outbreak size (grey line and ribbon). All values are shown on a pseudo-logarithmic scale (base 10 transformation), with tick labels in original case counts. Estimates for 2025 are excluded due to instability associated with a single sequenced case.

Estimates of outbreak size based on phylogenetic tree lengths and substitution rates of the dominant lineage (n=148) suggest that the true scale of the epidemic substantially exceeds surveillance data. The total outbreak size was estimated at approximately 28,637 cases, indicating that routine surveillance captured only 1–2% of infections annually (Figure 5). Early case counts remained relatively stable, further supporting extensive under-ascertainment of infections. Annual case counts were affected by long branches in pruned trees, suggesting that sequential pruning may be limited in resolving extensive unsampled diversity with deep ancestral origins. However, estimates derived from the full phylogeny remain robust to this effect, as they integrate unsampled diversity across the entire tree structure.

### Persistent local transmission is concentrated in foci with predictable directional spread

**Figure 6.**
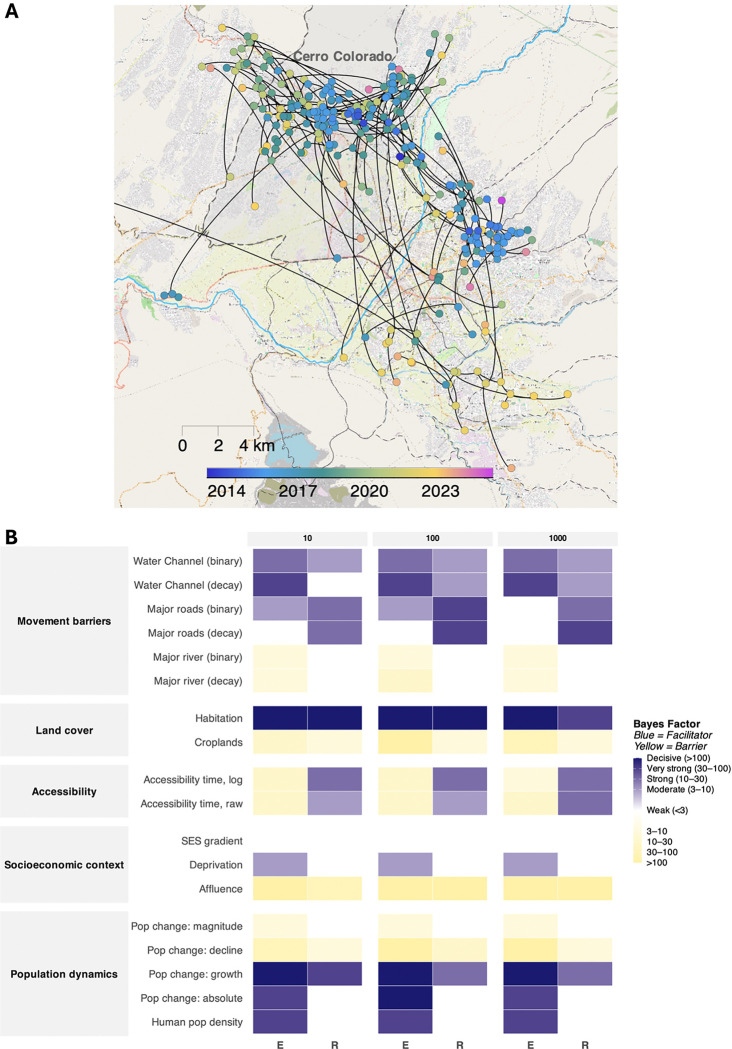
Statistical support for the association between landscape variables and rabies virus spatial diffusion in Arequipa city. (A) Continuous diffusion relaxed random walk (RRW) phylogeographic reconstruction for the main Arequipa lineage (n = 148 genomes) inferred in BEAST. Tip movements are overlaid on an OpenStreetMap base layer to provide environmental context and illustrate urban heterogeneity. District boundaries are shown as dashed lines, and the Cerro Colorado district is highlighted in light grey to indicate its role as a putative movement hub. (B) Summary heatmap of Bayes Factor (BF) support for associations between landscape predictors and viral diffusion, as represented in panel (A). Variables are grouped into resistance (barriers; yellow) and conductance (facilitators; blue) surfaces. Colour intensity indicates the strength of statistical support across a logarithmic BF scale, ranging from weak to decisive evidence. Analyses distinguish (E) the tendency of lineages to remain in particular environmental conditions and (R) the tendency to move toward them, across socio-ecological, environmental, and accessibility-related predictors.

Continuous phylogeographic reconstruction of the dominant lineage (Figure 6A) estimated a mean weighted diffusion rate of 4.15 km/year (95% HPD: 3.63–4.68 km/year) with a weighted diffusion coefficient of 6.82 km^2^/year (95% HPD: 5.72–7.72 km^2^/year). In contrast, unweighted estimates were higher (velocity: 8.09 km/year; diffusion coefficient: 8.40 km^2^/year), indicative of episodic long-distance dispersal events alongside local transmission. A weak isolation-by-distance signal was observed (r ≈ 0.19–0.20), indicating spatial structuring.

**Figure 7.**
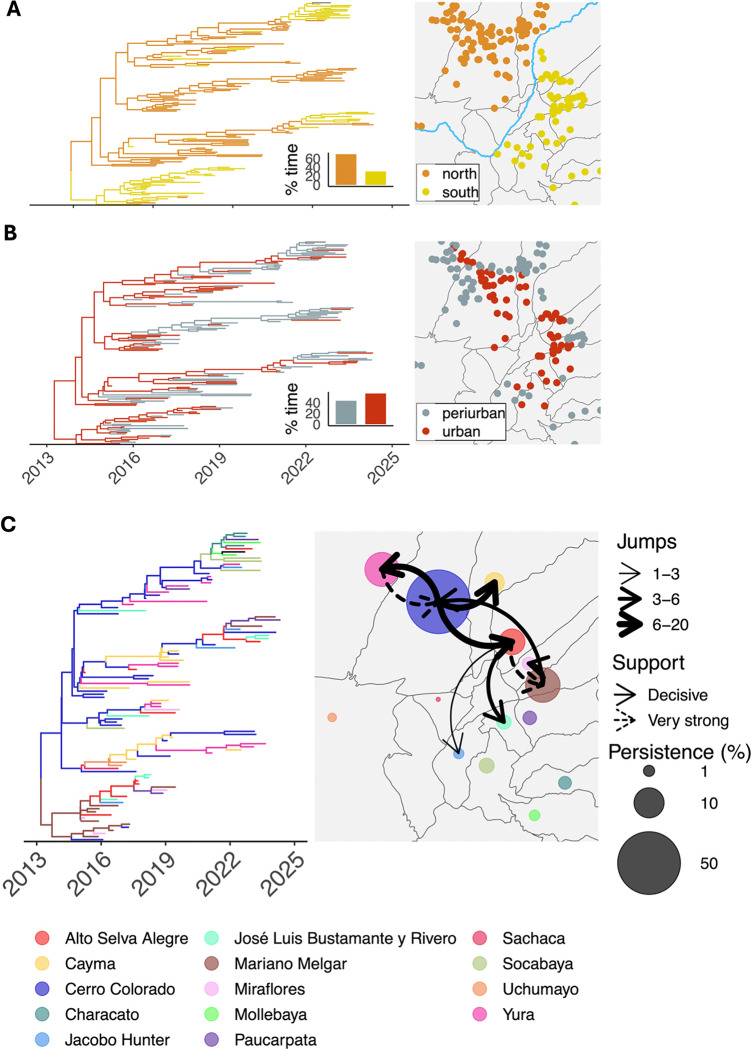
Discrete trait phylogeographic reconstruction of rabies virus transmission in Arequipa. (A) River-structured phylogeography across the Chili River (north vs south). Branches are coloured by inferred state. Inset shows Markov reward estimates of state persistence (percentage of evolutionary time). Map displays spatial distribution of cases. (B) Urban–periurban transmission structure, with phylogeny and map coloured by inferred urbanicity. (C) District-level transmission network and phylogeny. Branches are coloured by inferred district state. The map depicts inferred viral movements between districts (Markov jumps); arrow widths reflect Markov jump estimates, and line types indicate Bayesian stochastic search variable selection (BSSVS) statistical support for the route. Node sizes represent Markov reward (proportion of evolutionary time spent in each district). Only strongly supported transitions (Bayes factor > 50) are shown.

Discrete phylogeographic analyses (Figure 7) revealed strongly structured transmission across scales and movement was consistently asymmetric, with specific regions acting as net sources and others as net recipients. At district level, transmission was highly directional and spatially structured, with repeated preferential routes (e.g., Cerro Colorado to Yura and Cerro Colorado to Cayma) accounting for a disproportionate share of movement, while reverse transitions were comparatively rare (e.g., Yura to Cerro Colorado: 3–4 Markov jumps). Cerro Colorado emerged as the dominant transmission hub (highlighted in Figure 6A and 7), accounting for 51% of total evolutionary time and contributing most outgoing lineage movements (38 Markov jumps). Although multiple transmission pathways were statistically supported (17 transitions with BF > 10, including 7 decisive), only a subset contributed substantially to viral spread, indicating that transmission is concentrated along frequently used routes. Most supported transitions were robust to downsampling (Table S2), suggesting that they reflect underlying transmission structure rather than sampling artefacts. However, 3 of the 17 statistically supported transitions (all BF < 20) showed some sensitivity in downsampled datasets. For clarity, only transitions with BF > 50 are shown in Figure 7; all supported transitions are reported in Table S3.

This spatial structure was consistent across broader divisions. Lineages were predominantly located north of the river (69% of total evolutionary time vs 31% south), with bidirectional movement but a bias towards north-to-south transmission (12 vs 4 Markov jumps). Similarly urban areas acted as the primary foci (57% of evolutionary time), with substantially higher transmission from urban to periurban regions (mean outgoing Markov jumps = 45 vs 12 incoming).

Although no predictor was associated with lineage velocity (Q) (results not shown), landscape phylogeographic analyses identified several factors associated with viral persistence (E) and movement (R). Lineages were strongly associated with inhabited areas and higher human population density (decisive BF support). Notably, relative population change (but not absolute population change or population density) emerged with strong support for facilitating dispersal, suggesting preferential movement towards expanding peri-urban areas.

Socioeconomic structure had a pronounced effect on transmission. Affluent areas consistently acted as barriers to both lineage occurrence and dispersal, whereas deprived areas were associated with increased persistence. Reversing the socioeconomic resistance surface (i.e. switching affluence and deprivation as the high-resistance category) did not produce symmetric effects on connectivity metrics, indicating that landscape configuration and the spatial clustering of socioeconomic classes differentially constrain diffusion processes rather than behaving as simple inverses. Representing socioeconomic status as a continuous gradient yielded weaker and less consistent effects.

Anthropogenic infrastructure influenced movement. Major roads showed strong support for facilitating dispersal, while accessibility (travel time) acted as a barrier, indicating that connectivity shapes movement pathways. Water channels were associated with increased lineage occurrence but showed weaker effects on directed movement. Rivers exhibited moderate support as partial barriers, constraining but not preventing dispersal.

## Discussion

Our analyses reveal that rabies persists in Arequipa through highly structured local transmission shaped by interacting ecological, infrastructural, and socioeconomic factors. A single viral lineage has dominated transmission over nearly a decade, while occasional introductions have resulted in limited onward spread. Within the city, transmission is concentrated in a few interconnected urban districts, forming persistent hubs that structure dispersal. Roads, water channels, and heterogeneous urban development facilitate spread across otherwise separated neighbourhoods, while the Chili River acts as a partial barrier.

Arequipa RABV belong to the Cosmopolitan AM5 subclade that circulates across Peru, Bolivia, Argentina, and Brazil^30,57^. Despite ongoing regional connectivity, a single successful introduction has dominated transmission within Arequipa city for nearly a decade, consistent with observations from other endemic settings, including Harare, Zimbabwe^12^ and Gauteng Province, South Africa^58^. Although this lineage accounted for 97% of sampled viruses, we identified multiple independent introductions, including from Bolivia and Puno, and others likely occurred but remained undetected. While most introductions were epidemiological dead ends, at least one established sustained transmission, resulting in the 2021 El Pedregal outbreak^30^. Shared ancestry, a recent introduction from Bolivia, and confirmation of viral export from Arequipa to El Pedregal^30^ demonstrate continued epidemiological connectivity within the region. Bolivia has been repeatedly implicated as a source of canine rabies in Latin America^59^, where comparatively fragile control programmes have contributed to persistent transmission^60^. Together, these findings are consistent with rabies metapopulation dynamics, in which local transmission is sustained by occasional long-distance dispersal events^13,16,24,61–63^. Although regional connectivity has likely declined with improving control measures, our results highlight the need for coordinated surveillance and higher-resolution genomic data to detect and resolve these rare but epidemiologically important events.

We infer multiple years of cryptic transmission prior to first detection, and outbreak size estimates indicate substantial under-detection. These findings highlight a limitation of case-based inference in elimination settings: when incidence is low and transmission spatially patchy, reported cases cannot reliably distinguish elimination from ongoing (undetected) circulation. Historical eradication efforts, most notably smallpox, illustrate that verification of elimination requires intensive, multi-layered surveillance rather than reliance on case reporting alone^64^. Genomic epidemiology provides essential resolution and can reconstruct transmission pathways when routine surveillance is incomplete.

Within Arequipa, transmission is highly structured, with a few districts disproportionately sustaining spread. Rather than diffuse circulation, viral movement is concentrated along corridors linking several foci. Cerro Colorado emerges as the dominant hub, with neighbouring districts (e.g. Yura) forming part of a highly connected network. Both areas have previously been identified as rabies hotspots^39^, characterised by socioeconomic disadvantage, reduced access to health services, and rapid urban expansion from migrant labour^30,65–70^.

Landscape analyses revealed that multiple sociological and spatial factors are associated with lineage movement and persistence. Although each variable was evaluated independently within the analytical framework, the collective patterns suggest that rabies transmission is shaped by interacting social, demographic, and environmental processes. Population and socioeconomic characteristics appear to determine where transmission is maintained, whereas the urban landscape more strongly influences how the virus moves between these areas. Affluent neighbourhoods tend to impede spread, which may reflect greater confinement of owned dogs and higher vaccination coverage^71^, while more deprived communities may sustain transmission through larger populations of loosely owned or free-roaming unvaccinated dogs. Likewise, densely populated urban cores act as persistent transmission foci, whereas rapidly expanding peri-urban areas facilitate dissemination and the establishment of new transmission chains. Geographic features primarily influenced connectivity, with major roads facilitating dispersal while increasing travel time acted as a barrier, consistent with movement along established transport corridors. Although similar patterns have been reported at broader spatial scales^17,20,72^, our analyses demonstrate these effects operating at a much finer intra-urban scale. The complexity of the transmission dynamics identified here reinforces calls for systems-oriented approaches that combine urban planning, dog population management, vaccination, and public health interventions^11,73^.

Our findings provide important context for interpreting how urbanicity structures disease dynamics across a spectrum of urban forms, from urban–peri-urban interfaces to emerging secondary cities (Pedregal). Latin America is the world’s most urbanised region, and cities such as Arequipa have undergone rapid expansion over recent decades, accompanied by increased migration, informality, and inequality^65,67^. This has been associated with increased disease prevalence^30,65,66^. At a coarse scale, viral movement was predominantly urban-to-peri-urban, although this pattern should be interpreted cautiously because binary urban/peri-urban classifications fail to capture the heterogeneity of urban systems^73^. Our landscape analyses at much finer spatial resolution suggest that transmission is structured more by intra-urban connectivity than by broad urbanisation gradients. In particular, the micro-heterogeneous urban landscape of Arequipa, as captured by the microred health structure (sub-district operational units that reflect the organisation of community healthcare) may better represent the social and spatial pathways shaping rabies transmission than coarse binary classifications or conventional administrative boundaries, and should therefore inform future epidemiological analyses and control strategies.

The apparent predominance of urban-to-peri-urban viral movement should be interpreted with caution, as previous studies have proposed that peri-urban settlements, including populations of free-roaming and so-called “cave” dogs, act as reservoirs maintaining transmission within the city^74^. In addition, limited sampling of peri-urban dogs may have biased estimates of transmission directionality. Although truly feral dogs typically represent only a small proportion of dog populations in most settings^75,76^, the cave-dog ecology described in Arequipa may be a localised exception^71,74^. Lower vaccination coverage, greater roaming ranges, higher dog densities, and scavenging behaviour suggest that peri-urban dogs may contribute disproportionately to local transmission^38,71,74^. However, their contribution remains difficult to quantify with current sampling, highlighting the need for targeted surveillance to better resolve their epidemiological role.

This study has several limitations. Although we generated the largest genomic dataset for canine rabies in the region, sampling remains incomplete and uneven both within Peru and across neighbouring countries, with only 32 publicly available genomes from outside Peru. This level of sampling is comparable to that of other whole-genome phylogeographic studies of RABV^13,17^, reflecting the broader scarcity of genomic data for this pathogen. Nevertheless, these limitations reduce the resolution with which transmission pathways, directionality, and source-sink dynamics can be inferred. Although continuous phylogeographic approaches are generally less sensitive to sampling bias than discrete models^50,77–79^, residual bias, particularly from systematic under-sampling (e.g. lack of cave-dog samples) cannot be excluded. Occasional long branches in the phylogeny, some spanning approximately 50 transmission generations, are also consistent with unsampled transmission. Sensitivity analyses nevertheless indicated that key phylogeographic results were robust to downsampling of overrepresented areas.

Sparse spatiotemporal sampling also limits source attribution. For example, sample 219_2024 clustered with sequences from Argentina, Bolivia, and Brazil, but epidemiological evidence, travel history, and the recognised role of Bolivia as a source of canine rabies are most consistent with a Bolivian origin. Similarly, sample 75_2021 clustered with historical sequences from Puno, but the absence of contemporary WGS prevents distinguishing between a recent introduction and persistence within an unsampled transmission chain. Since the Puno Region has maintained zero cases of canine and human rabies since 2023^80^, this uncertainty raises the possibility of undetected circulation and highlights an important priority for surveillance. Genomic epidemiology provides a powerful framework for resolving transboundary transmission, but its effectiveness for elimination programmes depends on more geographically representative sequencing. This need aligns with regional coordination through PAHO’s Cooperation Among Countries for Health Development (CCHD) initiative, in which Peru and Bolivia are strengthening joint surveillance and control activities, presenting an opportunity to improve genomic capacity.

## Conclusion

Our findings have three main implications for rabies elimination in urban endemic settings. First, case counts alone are insufficient to infer elimination. We identify a long-lived lineage, a period of undetected circulation prior to first detection, and surveillance capturing only ~1–2% of infections, all indicating substantial cryptic transmission. Genomic data are essential for distinguishing introductions from ongoing undetected circulation. Second, transmission is highly spatially heterogeneous, requiring targeted control. Persistence and spread are concentrated in a few urban hubs and along key corridors linking urban and peri-urban areas. Control strategies should intensify intervention in these high-risk locations. Third, elimination is constrained by continued regional connectivity resulting in recurrent introductions, including at least one that caused onward transmission, requiring sustained control beyond the city’s boundaries. Overall, elimination in Arequipa requires integrated control across scales: improved surveillance to detect cryptic transmission, targeted interventions in hotspots and connectivity corridors, and coordinated regional control to reduce reintroduction risk.

## Supplementary Material

Supplementary Information

Supplementary text, tables and figures are available in the Supplementary Information file.

## Data Availability

Consensus genome sequences generated in this study will be deposited in GenBank prior to publication, with accession numbers to be provided upon submission. All code, analysis workflows, and supporting data required to reproduce the analyses are available at: https://github.com/kirstyn/arequipa-rabies-landscape-genomics
